# Experimental Models of Brain Ischemia: A Review of Techniques, Magnetic Resonance Imaging, and Investigational Cell-Based Therapies

**DOI:** 10.3389/fneur.2014.00019

**Published:** 2014-02-19

**Authors:** Alessandra Canazza, Ludovico Minati, Carlo Boffano, Eugenio Parati, Sophie Binks

**Affiliations:** ^1^Cerebrovascular Diseases Unit, Fondazione IRCCS Istituto Neurologico “Carlo Besta”, Milan, Italy; ^2^Scientific Department, Fondazione IRCCS Istituto Neurologico “Carlo Besta”, Milan, Italy; ^3^Brighton and Sussex Medical School, Brighton, UK; ^4^Neuroradiology Unit, Fondazione IRCCS Istituto Neurologico “Carlo Besta”, Milan, Italy; ^5^Brighton and Sussex University Hospitals NHS Trust, Brighton, UK

**Keywords:** brain ischemia, brain stroke, animal models, middle cerebral artery occlusion, magnetic resonance imaging, neuro-reparative therapies, stem cells

## Abstract

Stroke continues to be a significant cause of death and disability worldwide. Although major advances have been made in the past decades in prevention, treatment, and rehabilitation, enormous challenges remain in the way of translating new therapeutic approaches from bench to bedside. Thrombolysis, while routinely used for ischemic stroke, is only a viable option within a narrow time window. Recently, progress in stem cell biology has opened up avenues to therapeutic strategies aimed at supporting and replacing neural cells in infarcted areas. Realistic experimental animal models are crucial to understand the mechanisms of neuronal survival following ischemic brain injury and to develop therapeutic interventions. Current studies on experimental stroke therapies evaluate the efficiency of neuroprotective agents and cell-based approaches using primarily rodent models of permanent or transient focal cerebral ischemia. In parallel, advancements in imaging techniques permit better mapping of the spatial-temporal evolution of the lesioned cortex and its functional responses. This review provides a condensed conceptual review of the state of the art of this field, from models and magnetic resonance imaging techniques through to stem cell therapies.

## Introduction

Stroke is the second cause of death worldwide (1). Thrombolysis with recombinant tissue plasminogen activator (rt-PA) reduces mortality and morbidity from ischemic stroke, but is only available for around 5% of patients due to factors including necessity of administration within 4.5 h of lesion onset (1–3). The impact of new data proposing benefit for some patients of rt-PA within 6 h remains to be defined (4). In recent years, several avenues showing promises in pre-clinical research have failed to reach clinical viability and the need for new therapeutics is pressing (5).

Encouraging results have been accumulating in the domains of stem cell therapy and growth factors (6, 7). These results should be interpreted in light of systematic reviews identifying a lack of consistent rigor in study design and over-representation of positive data in the pre-clinical animal and stem cell literature (8, 9). However, investigational strategies can be enhanced through the development of superior animal models of stroke and advances in multi-modal magnetic resonance imaging (MRI), which offers a non-invasive method of studying tissue microstructure, perfusion, and functionality *in vivo*.

This review aims to provide a concise account of current experimental approaches in ischemic stroke. The main animal models will be recapitulated, before introducing recent advances in experimental MRI, with a focus on diffusion-weighted imaging (DWI), perfusion-weighted imaging (PWI), and functional MRI (fMRI). Finally, emerging stem cell therapies are considered with emphasis on explanation of relevant features for understanding and designing translational research.

## Experimental Models of Brain Ischemia

### Which models are available?

Human ischemic stroke usually results from middle cerebral artery (MCA) occlusion, and so techniques which occlude this artery are closest to the clinical picture (10, 11). Since open surgical methods of MCA occlusion were first described in the 1970s and refined in the 1980s (12–15), a number of additional techniques have been proposed, some of which avoid craniotomy (11, 16): the intra-luminal suture model (17–20) is commonly used (identified by Howells et al. (20) as the method employed in >40% of 2,582 neuroprotection experiments) but the thromboembolic model (21, 22), the coagulation or ligation model (17, 23), the endothelin-1 model (20–25), and the distal artery compression model (26) provide alternatives. Here, a selection of the principal models is introduced; for comprehensive details on those of interest and other models, the reader is further referred to recent in-depth reviews by Howells et al. and Krafft et al. (20, 27).

#### Intra-luminal suture model

**Figure 1:**
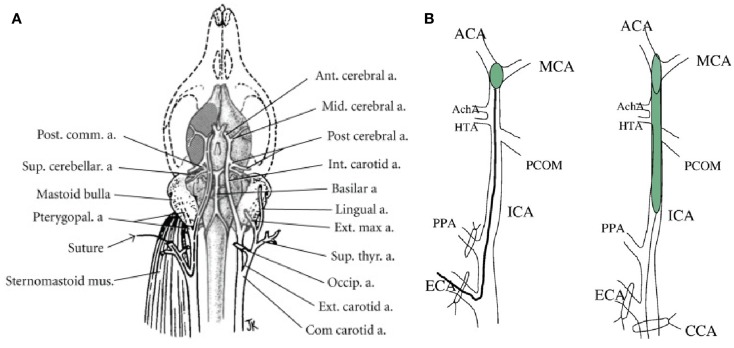
**(A)** Vascular brain anatomy of the rat. Reproduced from (10), by permission of American Heart Association Inc/Wolters Kluwer Health. **(B)** Induction of middle cerebral artery occlusion (MCAO) by insertion of thread into the external carotid artery (Longa’s method, left) or common carotid artery (Koizumi’s method, right). ACA, anterior carotid artery; AchA. anterior choroidal artery; CCA, common carotid artery; ECA, external carotid artery; ICA, internal carotid artery; HTA, hypothalamic artery; PPA, pterygopalatine artery; PCOM, posterior communicating artery. Reproduced from Ref. (23) by permission of Elsevier Inc.

This model was developed in the rat by Koizumi in 1986, modified by Longa et al. (10, 28) (Figure 1) and later refined by Kamii and Lo for application in mice (29, 30). A surgical filament introduced via the internal carotid artery (ICA) reaches and occludes the MCA at its origin (31). The technique may provide a model of transient ischemia with reperfusion or permanent occlusion according to whether the suture is left in place for 30 min to 2 or 24 h. Importantly, this technique does not require craniotomy which introduces extra-lesional influences on intracranial pressure and temperature (31). This relevance to clinical application is accentuated since in human subjects, surgical decompression post-stroke has been shown to extend survival through alleviation of cerebral edema (20, 32). The intra-luminal suture model is well-established in rat and mouse, but it is notable that lengthy, careful practice remains necessary to avoid complications, such as core temperature fluctuation, damage to neighboring structures, and hemorrhage, all of which can compromise experimental accuracy (31).

One controversy regards the extent of tissue damage with this model: there is evidence that, compared to the electrocoagulation technique, this model induces a larger ischemic lesion that exceeds the territory of the MCA to reach the thalamus, hippocampus, and substantia nigra. This might be the result of an additional effect on posterior carotid artery (PCA) flow, exerted by the intra-luminal thread, as it ascends the ICA (17). Many aspects including thread size and length, animal weight and strain, and exact filament positioning may contribute to vascular phenomena outside the MCA in the intra-luminal model (18, 20). Similarly, a direct comparison of the intra-luminal method or craniotomy plus MCA ligation to perform MCA occlusion indicated that the surgical precision of ligation was able to achieve most stable infarct volume and behavioral impairment (17). As the intra-luminal model is susceptible to issues of anatomical accuracy of the induced damage, a key consideration when implementing this model is the reproducibility of infarct volume, which may vary between centers. Despite these variables, the intra-luminal filament model is widely considered adequate to reproduce primary ischemic insult and subsequent neuronal cell death, glial activation, and blood brain barrier (BBB) damage and skilled practitioners may achieve high rates of experimental success (20, 33). Moreover, the presence of a significant ischemic penumbra early after occlusion makes this technique suitable for neuroprotection studies.

#### Thromboembolic models

These models may represent more closely the pathophysiology of human ischemic stroke and offer potential to test thrombolytic agents (34), evaluate ischemic lesions after thrombolysis (35) and study combination therapies, such as thrombolytic agents combined with neuroprotective drugs (27, 36). Early versions employed a human blood clot or suspension of homologous small clot fragments (37). However, they induced infarcts wherever spontaneous recanalization took place, and subsequent development of a technique of embolization of an autologous clot came closer to human arterial thrombi (38). This model remains widely used in rats and mice (20). Some researchers have described a model of embolic stroke employing microspheres which has been stated to reduce variability in lesion development and represent more closely the evolution over time and multifocal and heterogenous nature of human disease, albeit with the limitation of forming an irreversible insult (39, 40). However, a definitive microsphere model remains to be defined, as recently Zhu et al. documented at least one half of microspheres <100 μm failed to lodge in the rodent cerebral arterial circulation (41). This model is not suitable for reperfusion studies but avoids the complication of clot autolysis in clot models.

Excellent reproducibility is offered by the thromboembolic stroke model, in which injection of purified thrombin to the MCA can be performed with good precision (42). This model was previously given poor consideration in neuroprotection studies after repeated failure in translating animal results to humans. Moreover, the supra-physiological thrombin content in clots generated by this method may confer resistance to currently available thrombolytic agents beyond what is evident in the clinical picture (43). Yet despite perhaps greater accuracy of mechanical models, these do not reflect hemodynamic characteristics of blood reperfusion or post-lesional cerebral metabolism, factors influencing the response of brain tissue to neuroprotective agents (24).

#### Endothelin-1 model

This model achieves reversible occlusion of the MCA through direct or nearby application of the potent vasoconstrictor endothelin-1 (ET-1) (21, 22). It can be well reproduced in rats, offering benefits of an absence of lesions at injection site and low mortality, including in older animals, which is highly relevant to the human epidemiology of stroke (44, 45). Other strengths are its low invasiveness and its ability both to facilitate anatomical targeting of the lesion and to be implemented in the absence of anesthetic agents (20, 22, 23), some of which may attenuate stroke-induced morbidity in small animal models through potentially neuroprotective effects of hypothermia (20). It produces a dose-dependent stroke intensity in direct application models (21), but it is important to note a fourfold increase in dose requirement to achieve infarct in anesthetized rats (46); moreover, the potential for interaction of ET-1 with key molecular players in the pathological cascade or compounds under investigation must be considered (20, 22). It is not effective as a sole agent in mice, in which species it must be combined with another form of occlusion (23, 47).

#### Cerebral artery occlusion through electrocoagulation or mechanical methods

This group of techniques requiring open surgical access includes the electrocoagulation-surgical model described by Tamura and colleagues in the early 1980s, since which time a number of variations on this approach has been published (13, 48). While experienced surgeons can attain excellent reliability and low mortality with Tamura’s original method, disadvantages include the high level of expertise necessary and the production of a fixed deficit which inhibits study of thrombolytic therapies (20, 48).

Other mechanical methods of occlusion do permit the benefit of reversibility, such as use of an adjustable surgical ligature (49) or micro-aneurysm clips (50). These techniques also enable the application of ischemia over a defined time period, although both techniques – especially the use of clips – demand technical mastery, while to date creating a less predictable experimental lesion than Tamura’s approach (48–50). A more recent alternative is the transient distal middle cerebral artery occlusion (dMCAo), devised by Morancho and colleagues in 2012 (26). This model consists of compressing the distal portion of the MCA for 60 min using a blunted needle, followed by 24 h of reperfusion. Even though exposition of the left lateral skull and M1 portion of the brain are required, in many aspects this model is less invasive than the intra-luminal filament technique: certainly, vascular structure is well preserved, though this is to the detriment of inflammation induced by microglia even before the beginning of the ischemia. The lesion volume and mortality are comparable with the well-established permanent ischemia models, and this model deserves further investigation to better characterize the exact mechanisms of damage and recovery.

### Which species?

#### Small animal models

According to the stem cell therapies as an emerging paradigm in stroke (STEPS) guidelines (51, 52), an optimal pre-clinical stroke model should reflect human epidemiology, incorporate thorough monitoring of parameters of stroke development and collect robust safety data. Compared to larger species, small animals are less expensive, have easier husbandry and handling conditions, are more suitable for ischemic surgery and genetic modification, and are more ethically acceptable (23, 27). STEPS highlights the availability of proven rodent stroke models; in addition, the rat fulfils a key STEPS requirement of reproducibility of results (23, 51, 52), its cerebrovascular anatomy and physiology offers good similarity to that of humans (11) and it is of practical size for physiological monitoring and study of neuropathology via dissection or imaging modalities (19, 23).

Nonetheless, much interest has arisen in murine models of ischemic stroke, primarily focused on advances in genetic modifications permitting the modulation of specific molecular pathways in transgenic animals (53–55). Of note, ischemic cell death accumulates more rapidly in mice than rats, such that a 30 min MCAo induces a lesion equivalent to that produced within 60–120 min in rodents, while beyond this time the cerebral hemisphere is quickly infarcted (56). Additionally, there may be considerable inter- and intra-strain variability; for example, some transgenic mice lack communicating arteries within the Circle of Willis, notably the posterior communicating artery, which affects the extent of ischemic damage resulting after experimental intervention (57, 58). However, the challenge of differing cerebrovascular organization may also be found in some strains of rat (20). Other plausible factors are differences in arterial collaterals and sensitivity to excitotoxic cell death (56). Post-infarct temperature is a critical variable in both rats and mice; infarct onset may be complicated by the onset of hypothermia, which is thought to exert beneficial effect and is under debate as a clinical strategy (20, 56, 59), while hyperthermia exacerbates neurological deficits and neuropathology in rats (60). Similarly, fever is reported to have a deleterious influence over ischemia in a variety of clinical scenarios (20, 59, 60).

#### Larger species

Successful models have been developed for a wide range of species, including primates, pigs, dogs, cats, and rabbits. Larger animals have better similarity to humans in terms of behavior and sensorimotor integration, and STEPS and Stroke Therapy Academic Industry Roundtable (STAIR) guidelines agree it is necessary to test a positive result from a small animal drug study in a higher species prior to clinical evaluation in humans (51, 52, 61). In particular, such studies are necessary for pharmaceutical evaluation, including delivery and dosing of new compounds, which cannot be reliably derived from smaller animal models (51, 52, 61). In many aspects, larger animals offer a closer model of human cerebral anatomy and organization, as represented by the ratio of neocortex to basal ganglia and also the volume of white matter, which is sparse in rodents but is more plentiful – nearer to human neuroanatomy – in non-human primates (20, 27, 48). This is of especial pertinence in the field of lacunar strokes since these lesions most often occur in the deep white matter of human subjects (27). In contrast to lissencephalic brains of mice and rats, those large species which, like humans, have gyrencephalic brains, may provide closer models of functional recovery (61, 62). However, an important difference between the cerebrovascular organization of humans and many larger species is that in the latter, anterior blood supply to the brain is via a network of anastomosing vessels (the carotid rete), and so experimental techniques in which access to and occlusion of the MCA is achieved intravascularly (e.g., intra-luminal suture) may rarely be implementable (20, 63).

In the late 1990s, a reproducible and controlled model of intracerebral hemorrhage (ICH) was realized in the pig. As well as a low cost, this animal is analogous to humans in a brain rich in gyri and white matter (64). More recently, Imai et al. have proposed a new model of focal cerebral ischemia in the miniature pig, thus facilitating reproducible ischemic damage affecting gray and white matter in a gyrencephalic brain of a smaller animal. Current limitations include the potential influence of atmospheric exposure on intracranial pressure, and impact of diathermy occlusion, which may have the accessory effect of causing local blood–brain barrier rupture, thus preventing the investigation of reperfusion (65).

Another new model is of canine embolic ischemic stroke, which might have superior validity in terms of the exact relevance to human pathophysiology. However, this model requires more detailed characterization as well as confirmation by other groups (66). In 2008, Boltze and colleagues developed a robust model of focal cerebral ischemia in the sheep, with advantages of some similarities within hematological values and blood groups but fewer ethical dilemmas and lower mortality than primates. For the authors, the major advantages of the sheep model lies in its potential to study long-term impact of new therapeutic approaches and safety and efficacy of autologous cell therapies, to which the low cell yield of small species presents a practical obstacle (67).

As well as the ethical and practical challenges delineated previously, the maturation time of larger species (12–18 months) contributes financial factors to their place in drug development studies, and indeed financial considerations have been proposed as an impediment to the greater use of older animals, more reflective of human epidemiology, in stroke studies (20). For experimental ischemic studies, therefore, small animal and in particular rodent models are currently and likely to remain the most appropriate and widely used initial investigation platform (51, 52).

### Role of behavioral and functional outcomes

Structural outcome remains an imperfect predictor of prognosis including long-term recovery post-stroke (61). Studies in humans have demonstrated a link between extent of tissue damage and degree of functional impairment, and lesion volume is proposed as part of clinical forecasting tools. However, it is acknowledged that long-term resolution of neurological deficits is not tied to lesion volume alone, with important influences including age, gender, and co-morbidities (68). Additionally, there is increasing recognition and understanding of the power of neuroplasticity in the repair of neurological deficits (69). Therefore, together with methods which effect reproducible neuropathology, behavioral, and functional measurements are an obligatory component of animal models if the rate of translation of experimental therapies to the clinic is to be improved (48, 51, 52, 61). Moreover, these aspects assess what is in the real-life setting the most meaningful impact to prospective patients of potential new treatments (70).

#### Behavioral tests

A crucial issue in the translation from experimental to clinical settings is endpoint choice: there are fundamental differences in functional recovery measures adopted for humans and animals, and these unavoidably contribute to the difficulty of replicating experimental results in patients. Several comprehensive reviews of behavioral tests are available (71–73). In brief, the relevant domains are motor function, sensory function, and cognitive processing; no “pure” test exists, and the various tests available probe different combinations of multiple domains. It is therefore vital to combine multiple assessments, performed in a highly controlled manner.

The Rotarod remains one of the most widely used tests, involving positioning the rat or mouse on a rotating cylinder spinning at varying rates. The time-to-fall is measured, and provides an indication of general motor function and specifically motor learning. Notably, this test has been employed so widely that a key advantage is the sheer number of studies available for comparison of results. By contrast, the Wire Hanging test is a purer evaluation of sensorimotor function: animals are trained to hang with the forepaws to a metal thread, and the time-to-fall is measured. Performance on this test is chiefly determined by sensorimotor co-ordination. A less stressful and more ecologically comprehensive test is the Adhesive Removal test, in which adhesive patches are applied to the forepaws, and the time-to-removal is measured. Typically, the adhesive on the unaffected hemisoma is removed first, and the one on the other paw is removed significantly later, due to weaker perception and motor performance difficulties. This test includes more “general behavior” elements than the Rotarod and Wire Hanging tests, and is performed in the animal’s cage. Grip strength measurements, though common in other areas, are seldom employed. On the other hand, very detailed data can be acquired through computer-vision gait analysis systems, but such systems may be prohibitively expensive (73–75).

Tests that tap more directly into general behavioral and cognitive function include the Open field test and the Water maze. The Open Field test involves positioning the animal in a large open arena (50–100 cm) surrounded by suitable walls, and quantify exploratory behavior based on landmarks such as squares or with automated movement tracking; both horizontal and vertical (standing) activity is quantified. The Water Maze test involves placing a rat or mouse in a pool, where an escape platform is hidden just under the water surface; in presence of intact memory function, the platform position is quickly learnt from environmental cues. In presence of hippocampal damage, or sensorimotor impairment, the escape trajectory becomes less direct (76–78).

From a neuroanatomical perspective, the tests above probe different combinations of cortical and subcortical integrity and as such are complementary in respect to assessing functional impairment and recovery after experimental ischemia. In addition, some tests seem to be particularly suitable for the measurement of long-term deficits, as they avoid “ceiling” effects due to rapid recovery of baseline performance in the subacute phase; from this perspective, tests that tap into general behavioral integrity, such as the Open Field, Water Maze, and Adhesive removal tests appear to be more sensitive than more elementary ones such as the Rotarod and Wire Hanging tests (71, 73, 74, 77).

#### Functional recovery

Despite the many behavioral tests outlined above, an enduring challenge is the lack of a standardized single battery of tests, which might be used across the stroke research community for a particular animal model (48). Therefore, varying methods of functional evaluation are employed by different research groups. The situation is more pronounced in mice than in rats, due to a more established stroke research pedigree in the latter. Researchers are addressing this scenario and a range of evaluations able to delineate evolution of both short and longer-term neurological deficits in a murine transient occlusion model of ischemic stroke was recently announced (75).

Rodent and murine models have been developed which incorporate co-morbid factors such as old age and diabetes. Lindner et al. studied functional deficits in 24-month-old rats in which stroke had been induced using the intra-luminal suture method. An initial problem was a very high post-procedure mortality rate of 80% which, through utilization of a non-poly-l-lysine coated suture, the group overcame to produce a much lower 24% loss (79). Also using the intra-luminal suture model, Prakash et al. showed diabetic rats demonstrated both inferior re-vascularization and functional recovery to non-diabetic animals (80). One relevant point for consideration when selecting measures of functional outcome is that older animals may already exhibit baseline impairment or even inability to perform certain assessments such as beam-walking (79). It should also be considered that some techniques of producing ischemia may intrinsically damage measures of post-stroke recovery, for example, Tamura’s method which affects muscles of mastication and hence feeding (70).

Functional recovery in rodents has also been studied in other types of stroke induction, for example, the endothelin-1 and embolic stroke models (81, 82). Whereas in the embolic model, there was solid correlation of lesion size to functional neurological outcome (81), an interesting difference was noted between hemorrhagic and ischemic lesions of equal size in the endothelin-1 animals. This was posited to reflect differing neuroplastic phenomena in the different stroke etiologies, confirming that lesion size alone is an imprecise predictor of eventual neurodisability (82). A similar lack of correlation between lesion size and behavioral outcomes has been detected in mice, leading to a conclusion that lesion volume should not be considered in isolation, with functional assessment an equally vital measure in pre-clinical studies (75).

There are a number of acknowledged difficulties in applying results from small animal models to human subjects. Small species are noted to mask functional impairments (48). They recover more quickly than humans from neurological insult and harbor differences both in functional neuroanatomy, having a much lesser degree of lateralization of brain function, and structural neuronatomy, as outlined in Section “Larger Species” (48, 79).

On the other hand, primates more closely mirror both human pathophysiology of stroke and mechanisms of neural repair in several aspects including disease timeframe and cortical remapping of a brain also adapted for lateralization of function (69, 83). In addition, clinical manifestations of disease are rather similar; post-MCA occlusion, marmoset monkeys demonstrate contra-lateral weakness and neglect which parallels the deficits in human stroke patients. Moreover, functional tests in primates can more closely mimic and evaluate skilled motor tasks impacted in the human condition (84). An additional factor is that the larger central nervous system of primates facilitates imaging studies, although recent murine research employed the technique of optical intrinsic signaling which showed promise in the study of neuroplasticity in this species (84, 85).

## Magnetic Resonance Imaging

Traditionally, the first-line approach to evaluate the characteristics of stroke models and their response to therapeutic interventions has been histopathological and immunohistochemical analysis. While providing excellent structural detail and specificity, this approach has the inherent limitation of not allowing longitudinal examination, which is most important since the effects of therapeutic intervention on the spatial-temporal evolution of ischemic lesions are complex and need to be assessed at multiple time points, not just through an arbitrary “snapshot.” The need to track lesion progression in time, both structurally and functionally, has thus driven a shift from experimentation based on neuropathological analysis to non-invasive *in vivo* imaging-based analysis (86).

At the center of an ischemic area it is often possible to identify an irreversibly infarcted tissue core surrounded by a rim of acute oligemia, where energy metabolism is largely preserved but at the expense of severe tissue acidosis. This area, referred to as “ischemic penumbra,” is less severely hypo-perfused than the ischemic core and characterized by impaired neuronal activity without irreversible neuronal damage. Due to progressive metabolic failure the penumbra unavoidably evolves to necrosis without therapeutic intervention within a certain time window (87, 88). For this reason, the early identification and recovery of penumbra represents the principal aim of therapeutic strategies.

In the last 30 years, many imaging techniques have been proposed to detect and distinguish penumbra from the ischemic core. Positron emission tomography (PET) is still considered the gold standard to delineate the penumbra, given that it provides direct quantification of glucose metabolism integrity (89). Unfortunately, the availability of PET is limited, particularly for experiments on small animals that require dedicated micro-tomographs. Until recently, the role of MRI has been limited to structural imaging, particularly using T2-weighted sequences wherein very good anatomical contrast is shown between healthy and infarcted parenchyma. However, conventional structural MRI is hardly viable as a longitudinal imaging tool, since the lesion-related contrast changes become visible only at the time when penumbra has already largely converted to irreversible necrosis (90).

In the last decade, novel MRI techniques known as DWI and PWI have emerged, offering direct visualization of microstructural damage and perfusional alterations and thus opening new windows on the *in vivo* investigation of the pathophysiology of brain infarction. In parallel, the use of fMRI in animal models has gained ground, providing additional information on the integrity and plastic re-organization of neural responses in peri-lesional and contra-lateral areas (91).

### Diffusion- and perfusion-weighted imaging

**Figure 2:**
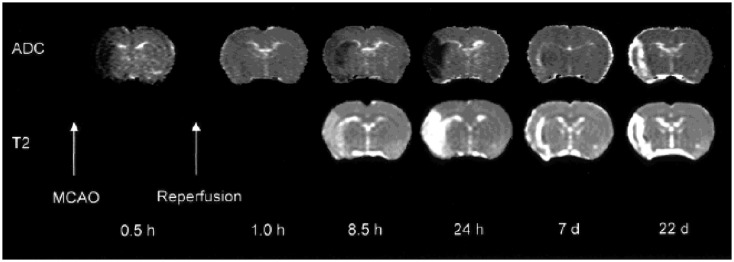
**Diffusivity and T2 maps demonstrating the consequences of transient left MCAO in the rat**. Time-courses of diffusion (ADC) and T2 contrast changes. Early ADC pseudo-normalization is followed by a further reduction, until eventually the ADC is significantly elevated with respect to tissue in the contralateral hemisphere and the two maps appear similar. Reproduced from Ref. (139) by permission of Wiley-Liss/John Wiley & Sons Inc.

Diffusion-weighted imaging reveals strong image contrast changes within minutes following the onset of acute cerebral ischemia and additionally allows the follow-up of tissue changes through the subacute and chronic phases (92, 93). During ischemic stroke, the apparent diffusion coefficient (ADC) follows a “U-shape” time-course, characterized by a rapid initial decrease, as early as 20 min after the onset of the ischemia (94, 95): intracellular water accumulation, due to rapid failure of high-energy metabolism and associated ionic pumps, leads to cellular swelling (cytotoxic edema) and consequent narrowing of the extracellular matrix volume (96). This is a robust effect that is consistently observed in experimental models (Figure 2) and patient studies. After 3–5 days, the ADC in the irreversibly infarcted area begins to increase, due to cell lysis and development of vasogenic edema; after a period of “pseudo-normalization” of ADC values, the lesioned area stabilizes on high diffusivity values. In particular, in acute phase ADC maps, when combined with perfusion measurements, allow to distinguish the core region from the penumbra, with ADC gradually decreased from the periphery to the center of the ischemic region (97–99).

On the other hand, PWI provides information on the hemodynamic status of brain tissue, delivering a multi-parametric representation of regional perfusional state of regions with impaired cerebral perfusion. In practice, PWI can be implemented using an exogenous paramagnetic contrast agent or using endogenous contrast through magnetic labeling of arterial blood. The first approach is the most widely implemented one, is least demanding in instrumental terms and provides very good measurement accuracy (100, 101). However, it is hampered by the need to have access to a suitably large vein to deliver a well-defined bolus. While in rats the tail vein is frequently chosen, in mice it is very small and collapses easily, so specific venous catheters are frequently utilized to establish access to the femoral vein. PWI delivers multiple parameters representing perfusional status, notably cerebral blood flow (CBF) and volume (CBV), time-to-peak (TTP), mean transit time (MMT) (95, 100, 101). These are interpreted synergistically to deliver a comprehensive picture of lesion status according to the following temporal progression model. After onset of ischemia, the ensuing vasodilator reaction first leads to an increase in CBV and MTT, while successfully regulating the CBF to normal values. A further fall in arteriolar pressure leads to an additional compensatory attempt, manifesting as a prolongation of MTT, which however fails to regulate CBF that begins to decrease. As the situation progresses toward local hemostasis, CBF falls drastically, the CBV paradoxically falls back to “normal” values and the MTT is elongated even further; at this point, the supply of oxygen and glucose is inadequate to support normal cell metabolism. Eventually, the CBV and CBF fall to zero and the MTT becomes un-measurable, at which point infarction has become irreversible. In light of these relationships and of the associated temporal progression, regions with a very low CBV can be taken as representative of the infarcted core, whereas areas with near-normal CBV and CBF but markedly increased MTT can be considered an estimation of the extent of the ischemic penumbra (102).

The spatial extent of perfusional deficits consistently exceeds the area of altered ADC, delineating a penumbral region where hypoperfusion is present but not severe enough to cause irreversible metabolic failure with consequent cytotoxic edema. The volumetric difference between these two areas, with near-normal ADC values but reduced CBF, is called “DWI/PWI mismatch.” The mismatch represents only a rough approximation to the real extension of penumbra, defined using the “gold standard” combination of PET with anatomical examination. There are multiple reasons why DWI and PWI can only approximate the penumbra extent, most notably the fact that DWI is only a very indirect measure of metabolic status, and the fact that there is no consensus on the optimal thresholds to use to delineate abnormal diffusion and perfusion. Further, thresholds optimized in one species or strain may not be applicable in others (97, 98, 103). While considerable progress has been made in recent years, the choice of DWI and PWI thresholds for penumbra delineation remains a severe issue also in human clinical trials (101, 104). Nevertheless the DWI/PWI mismatch is a convenient “surrogate” marker for the identification of the ischemic penumbra, since it represents an often-acceptable approximation to the real extent and does not require the use of PET to study glucose metabolism (105).

**Figure 3:**
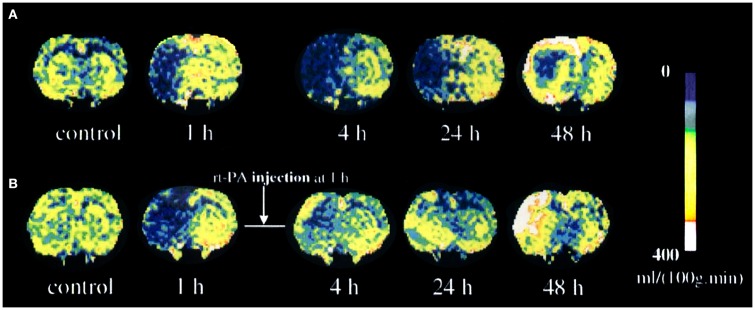
**Temporal evolution of cerebral blood flow (CBF) changes for untreated (A) and recombinant tissue plasminogen activator (rt-PA)-treated (B) rats after embolization**. Reproduced from Ref. (106) by permission of Lippincott-Raven Publishers Inc/Nature Publishing Group.

Unfortunately, mainly due to scanning time limitations, many research groups in the past have used only DWI or PWI alone for assessing therapeutic response in experimental models. However, those that do have obtained results clearly demonstrate the value of combining these techniques. For instance, Jiang et al. have demonstrated that rapid recanalization by means of rt-PA of embolically occluded brain arteries leads to increased rCBF and ADC which foreruns reduced lesion volume (106) (Figure 3). In another study, Meng et al. using DWI/PWI mismatch in a MCAO model demonstrated a reduction of volume of the infarcted lesion due to a mechanical reperfusion at 60 min after ischemia onset (107).

### Functional MRI

Rescuing the tissue in the ischemic penumbra is only one of the possible approaches to mitigate the functional consequences of brain stroke, and neuro-reparative therapies focus also on enhancing plasticity in the surrounding areas, and, more broadly, in the trans-hemispheric neural circuits affected by destruction of the lesioned cortex. It is therefore important to be able to study how functional responses evolve in the progression from acute to chronic phase. In clinical practice, fMRI has gained acceptance as a tool to evaluate activation patterns in response to motor and language tasks and, in stroke patients, the changes in activation patterns that follow stroke onset and gradual functional recovery have been explored in detail (108, 109).

In principle, fMRI has good potential for cross-translation of functional results between patients and animals. However in practice, its implementation in animal models is very challenging, mainly because of the near-impossibility of implementing active tasks that require effortful participation, limiting the usable paradigms to those based on passive stimulation. Despite obvious differences, with respect to active tasks, somatosensory stimulation paradigms have value because they do elicit significant activity in pre-motor and motor cortices in addition to primary somatosensory areas. Several studies have been published using such paradigms in rats and mice, essentially demonstrating that ischemic lesions in the sensorimotor cortex are associated to three changes: (1) loss of hemodynamic response in the infarction core, due to neural loss, and in the penumbra, due to metabolic dysfunction, (2) rapid emergence of increased contra-lateral activation, principally representing loss of trans-callosal inhibition, and (3) slow and gradual appearance of new ipsi-lateral activation clusters in peri-lesional areas, representing re-organization attempts to recuperate function through cortical plasticity (110–112).

**Figure 4:**
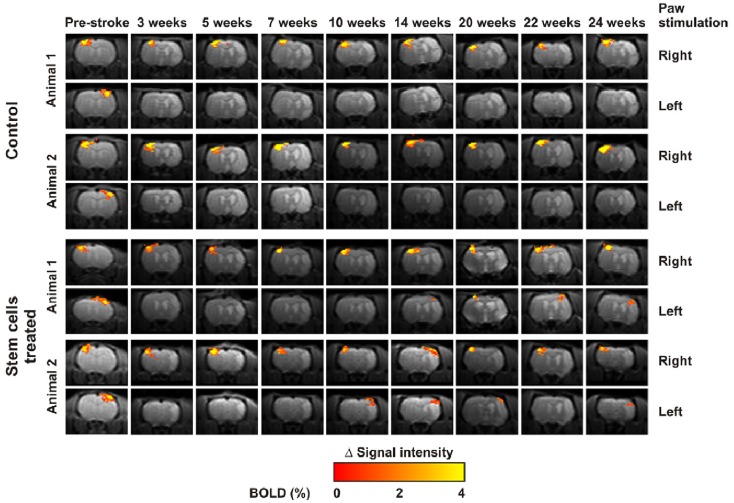
**Temporal changes of functional activation elicited by forepaw electrical stimulation over 6 months**. Rats treated with stem cells (bottom) demonstrated more rapid and marked “renormalization” of the activation pattern in comparison to controls (top). Reproduced from Ref. (114) by permission of the authors.

The complexity of these response patterns implies that there is no straightforward answer as to whether increased or decreased activity in a given region is a sign of functional recovery. Some studies have demonstrated that functional recovery is mainly associated with activation patterns that resemble as closely as possible those obtained prior to lesion onset (113) and that successful reparative therapies enhance this pattern of functional recovery (114) (Figure 4). For interpreting the nature of specific effects, correlation with behavioral scores is essential, to distinguish between loss of inhibition and functional specificity from genuine plasticity supporting functional recovery (115, 116). There are two main problems implementing fMRI in animal models. The first is anesthesia, which by definition suppresses central nervous system activity and for which there may be a very narrow dose margin available, implying that the dose cannot be lowered enough to obtain reliable fMRI responses without also incurring into movement artifacts. The other problem is that the hemodynamic response at the basis of fMRI can be impaired for countless reasons that are not related to neuronal function, such as impaired cerebrovascular reactivity. For this reason fMRI is frequently performed in conjunction with evoked potentials, which do not provide good spatial information but, on the other hand, represent neuroelectric activity directly and may therefore detect situations where neural activity is preserved but rendered invisible to fMRI through physiological confounds (116).

### Resting state functional MRI

An alternative fMRI method, variously referred to as resting state fMRI (rs-fMRI) or functional connectivity MRI (fcMRI), assesses spatial functional correlations within neural networks without the need of a stimulation paradigm. The notion is that of studying the temporal coherencies that spontaneously emerge from intrinsic brain activity and which remain manifest even under moderate levels of anesthesia. This method has several potential practical advantages, most notably that of not requiring active stimulation with the associated equipment complexities and movement artifacts. Analysis of rs-fMRI datasets demonstrates the presence of a rather well-defined set of stereotypical networks, which include a “sensorimotor” network that spans the motor and sensor strips bilaterally and extends to the pre-motor areas (117, 118). Recent work has demonstrated that such networks are clearly identifiable also in small animals and several groups are exploring the application of these techniques to study functional re-organization after stroke (119). Together with advanced network analysis, serial-state rs-fMRI was used by Van Meer et al. to identify mutually influential changes in gray matter functional connectivity, white matter structural integrity and network topology of the sensory and motor systems of both hemispheres and demonstrate impact on functional gains in rats following medium or large single hemispheric stroke (113).

### Cell tracking

A further emerging application of MRI is the tracking of delivered stem cells. Tracking the temporal progression of their diffusion from the site of administration is obviously of central importance for the development of reparative therapies, as much remains to be understood about the guiding mechanisms and how migration to the lesion site can be promoted. Until recently, the role of MRI was limited to tracking cells labeled with super-paramagnetic compounds. While some studies have demonstrated successful detection, substantial uncertainty remains regarding the ability to unambiguously detect migrating cells over and above the existing anatomical contrast and, especially, about the inherent toxicity that such compounds can have. A more recent approach is that of labeling the cells with 19F, which is not physiologically present and which can be detected with minimal modifications with MRI hardware. This approach retains the advantages of MRI, in that good anatomical contrast can be obtained through 1H imaging and no radioactivity is involved, but has major advantages because cell tracking signal is provided over an essentially zero physiological baseline, and because the potential toxicity issues encountered when embedding large doses of metallic compounds in stem cells are inherently avoided (120–123).

While there is a wealth of literature on MRI-based cell tracking for monitoring reparative interventions in neurovascular and oncological disease, there are fundamental issues that remain open and need careful consideration (124, 125). Although existing studies demonstrate that short-term tagging under experimental conditions is feasible, we still have a rather constrained understanding of the effect of tagging on biological viability, of the exact bio-kinetics of the tagging molecules and of long-term tagging stability. For example, a very recent study has demonstrated that iron oxide nanoparticles administered intravenously after MCAO, which can produce intense signal changes in the ischemic region, are not phagocytosed by blood-borne leukocytes and do not enter the ischemic mouse brain as previously believed (126). The authors highlight the issue as an example of publication bias in a controversial area, involving a potential “decoupling” between the beliefs of the majority of researchers, who are aware that certain tagging techniques have certain limitations, and the published literature, where positive findings are preferentially highlighted in the most influential journals.

Other issues that remain to be addressed carefully include that of false positivity due to label retention by dead cells and label uptake by “bystander” cells; further, while binary detection can be attainable, reliable quantification is extremely complex, because the relationship between cell density and signal changes can be highly non-linear, due to tracer dilution as well as to a range of endogenous contrast sources which can confound measurements, such as iron content in the basal ganglia and bone marrow (BM) (127). While short-term tagging can be highly informative in experimental conditions, translational applications will unavoidably rely heavily on long-term longitudinal assessments, the feasibility and reliability of which critically depends on long-term toxicity, stability, and retention, as well as on stability of intrinsic tissue contrast, all of which have been investigated only partially and may require recourse to large animal models to obtain a more definite characterization (128).

Future work will, in particular, need to assess comparatively the strength and weaknesses in terms of toxicity and stability of magnetic microparticles and fluorinated compounds; the biophysical differences are profound, and this reflects on the suitability of different marking strategies in experimental or translational applications (123). Notably, while MRI-based cell tracking can have a wealth of advantages in terms of availability of multiple anatomical and functional contrasts and ease of clinical translation, PET- and optical imaging-based cell tracking is feasible and can have substantial sensitivity advantages (129).

## Cell-Based Therapeutics

### The need for new strategies

Thrombolysis with rt-PA is the standard intervention in ischemic patients. Although this reduces disability if administered according to protocols, it carries a risk of ICH of 5.2%, as compared to 1% in controls (3). Moreover, many patients arrive outside of the therapeutic window and the time of onset of the ischemic insult may be unknown (1). Therefore new interventions are desirable to halt the ischemic damage and preserve undamaged brain regions. Since neuroprotective compounds showing promise in animal studies have yet to demonstrate efficacy in clinical trials (5) encouraging data from animal studies of stem cell-based therapy represents a valuable investigative avenue (53). Stem cell transplantation could be implemented weeks or months after injury, extending opportunities for therapeutic intervention (7, 130, 131). Indeed, there are positive expectations of human trials currently underway to explore stem cell administration in the long-term neuro-rehabilitation phase (130).

### Types of stem cell therapy

Different routes for the potential acquisition of stem cells exist; they may be obtained endogenously through mobilization of the patient’s own neural stem cells (NSCs), or exogenously in the form of a multipotent cell type, mesenchymal stem cells (MSCs) (1, 132). Exogenous sources of MSCs include embryonic material, certain adult tissues, e.g., BM (which also offers the opportunity of autologous grafting) and adipose tissue (133–135). One advantage is that MSCs do not express the major histocompatibility complex in their undifferentiated state, therefore the risk of tissue rejection at the time of implantation remains low. Therapy could be achieved through transplantation to the affected region of cells pre-differentiated *in vitro* (136), or through direct injection of stem cells differentiating *in vivo* into the needed cell type (137). There is evidence that stem cells migrate preferentially to injured areas and release factors promoting survival and regeneration in areas of high resident cell death (138). This may be the key mechanism by which such cells promote recovery, as suggested by a model of rodent ischemia, where transplanted murine NSCs were no longer viable at 6 months and could not create connections within existing brain tissue (114).

### Endogenous NSCs

In 2002, two research groups observed transient MCAo in the rat inducing expansion of neuroblasts from the ipsi-lateral sub-ventricular zone (SVZ); unexpectedly, these cells migrated in chains to the ischemic penumbra (140, 141) rather than their customary destination, the olfactory bulb. Some survived and displayed markers of mature striatal spiny neurons akin to those destroyed by the infarction, with new neurons added to the striatum for months (142, 143) or even a year (144) after stroke. There is a growing list of endogenous molecules involved in the regulation of adult neurogenesis, such as bFGF (basic Fibroblast Growth Factor), which promotes cortical cell replacement and functional recovery in neonatal (145) and adult (142) rats; EGF (Epidermal Growth Factor), a more potent augmenter of SVZ-derived cells in striatal tissue (146); and Nogo-A, a neurite inhibitor influential in murine models of ischemia and whose signaling cascade wields critical input in the regulation, maturation, and migration of NSCs from the SVZ (147, 148). However, debate continues over long-term functional improvement; in a murine model of stroke, animals in which blockade of endogenous neural stem cell production was inflicted suffered larger stroke volume and short-term worsening of performance on a number of behavioral measures, but at 12 weeks there was no difference in deficits between treatment and control groups (149). Investigation of factors in mobilizing NSCs should devote similar attention to their potential for unexpected adverse effects. Excessive stem cell proliferation is a well-known risk factor for genetic mutations and malignant transformation, and endogenous NSC mobilization is a young technology compared to use of MSCs, whose study stretches decades (150, 151).

### Exogenous delivery

#### General considerations

Advantages of exogenous stem cell transplantation include control over cell delivery, reducing risk associated with mitogen infusion, and cell fate control (152). An additional quality is the potential to genetically modify MSCs by means of a plasmid vector to express beneficial proteins. For example, erythropoietin-expressing BM-derived MSCs have successfully been introduced in a murine model of anemia, remaining biologically active for up to 5 weeks and providing an alternative to viral vectors, which may be associated with some safety concerns as well as challenges in devising a successful vehicle for delivery (153).

Technical considerations include whether administration should be via the vasculature (154) or directly into the brain (155). For example, BM-derived stem cells are more frequently delivered intravenously, whereas NSCs are injected directly into the brain parenchyma. In animal models of cerebral ischemia, improved cell survival and neuroprotection is seen with delivery to the penumbra compared to the ischemic core (156). Dharmasaroja reviewed three methods of MSC transplantation and differentiation: intrastriatal, intracarotid, and intravenous (IV), concluding capability of cells to reach the pathological site and positive effects by all three routes (133). Although intracerebral delivery of MSCs may additionally reduce lesion size, IV infusion also offers biological and functional benefits and may be more practical in a clinical setting (133) albeit permitting fewer cells to reach the central nervous system than more direct routes (132). IV administration seems to be the optimal route for chronic stroke treatment, as shown in small clinical studies where no major adverse effects were found (157). Nevertheless, intra-arterial (IA) delivery has shown higher cell engraftment in the brain compared to IV administration, but it requires further development since it produces new brain infarction in animal model studies (158).

#### MSCs from embryonic and adult niches

Drawing on evidence that BM cells may develop into a variety of neural cell lines (159, 160), Zhang and colleagues (161) injected BM mononuclear cells from healthy adult mice into the tail vein of mice with MCA coagulation. Cells attained the peri-infarct zone and transdifferentiated into putative cortical neurons within the first few weeks of post-experimental injury, adding to evidence for beneficial effects of BM cells in the treatment of experimental acute brain injury (161–165).

Wakabayashi et al. reported that IV transplantation of MSCs from the marrow of human fetal vertebrae significantly improved the neurological condition of rats 7 days after MCAo, which is suggestive of biological pathways outside of differentiation into neuronal cells and CNS integration (164). MSCs were undifferentiated in the ischemic core at 3 days and almost undetectable at seven, but they fostered reduction of infarct volume and functional recovery through graft-induced modulation of neurotrophic factors and beneficial cytokines in host cells. This is not the only paper demonstrating that transplanted MSCs remain undifferentiated. Tsai et al. noted a similar phenomenon, but although few MSCs differentiated to neural cell types they exerted a favorable effect on angiogenesis, probably through mediating release of neurotrophic factors by host cells. Of potential clinical relevance, pre-treatment with established mood stabilizers lithium and valproate had increased numbers of MSCs homing to the ischemic destination (165).

Functional benefits of stem cell therapies have been demonstrated in rodent MCAo models. In one study, IV delivery of MSCs from rodent BM achieved improvement in neurobehavioral outcomes of treated compared to control rats at 12 weeks, although few cells attained the central nervous system. These results were credited to positive changes in both the peripheral and central nervous system immunological milieu (166). Another study employed human umbilical cord blood-derived MSCs, given intrathecally or intravenously in a rodent MCAo model of stroke (167). Although functional progress was similar in both groups, more effective cell migration and neural differentiation and a greater impact on ischemic damage was observed with the intrathecal route. A barrier to clinical application in humans is the large number of cells required in both methods. Despite this, in light of their positive potential, further study of BM-derived MSCs is warranted to delineate their use in more detail, including long-term functional benefits, especially as autologously derived cells are relatively available and do not carry the ethical and immunological drawbacks of fetal material (133, 154).

#### Differentiation of human adipose cells

**Figure 5:**
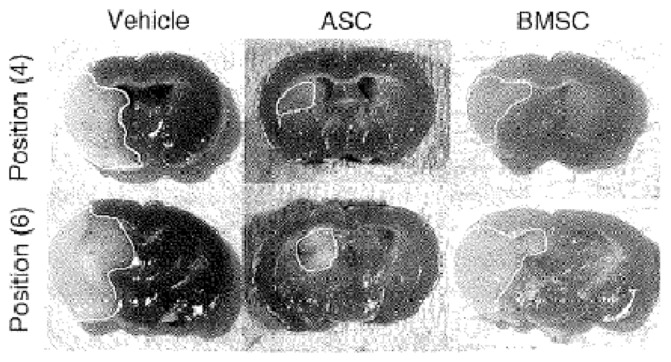
**Representative tetrazolium chloride strained sections of ischemic mice treated with vehicle (left panel), adipose stem cells (ASC, central panel) and bone marrow stem cells (BMSC, right panel)**. Reproduced from Ref. (168) by permission of Informa Healthcare Inc.

An alternative and plentiful source of adult MSCs is adipose tissue, which is accessible with a potential supply via liposuction procedures (134, 135). It poses fewer ethical challenges than embryonic sources and adipose-derived stem cells (ASC) may have greater potency in regenerative settings than other cell types. A murine study of ischemic stroke therapy highlighted a 1000-fold richer density of MSCs in adipose tissue compared to BM, and ASCs are known to expand more efficiently than BM stem cells of equivalent density and proliferation index (9, 168, 169). Ikegame and colleagues (168) found that after 48 h in culture, ASCs secreted higher levels of growth factors including hepatocyte growth factor (HGF) than BMSCs. *In vivo* experiments supported this ASC potential, demonstrating both improved functional performance and higher quantities of growth factors and smaller lesion size on neuropathological samples of ASC compared to BMSC-treated mice (Figure 5). As in other studies, the transplanted cells did not thrive as none were detected in histological analysis of the infarct at 24 h, again suggesting gains are due to trophic influences and even that transplanted cells may not differentiate conclusively into mature neural lineages (168, 169). In addition to positive experimental effects, since ASCs offer options of autologous harvesting and bear low potential for immunological reactions, their investigation as stroke therapy should certainly continue. However, long-term safety studies are vital before clinical implementation, as secretion of trophic factors and indeed tumorigenicity of MSCs may create risk of neoplasm (170).

### Evaluating the literature

Despite the promise and excitement of stem cell based therapies, the high risk of publication bias must be acknowledged, since it has been estimated as high a number as 1/6 of animal stroke studies are not reported. Moreover, not all pre-clinical studies employ robust experimental methods such as randomization and blinding to outcome, nor do they enroll animals reflective of human epidemiology – namely elderly patients with underlying health problems (8, 9). Lees et al. specified stringent inclusion criteria for a systematic review and meta-analysis of autologous and allogeneic (but not endogenous) animal stem cell studies in ischemic stroke, and concluded pre-treated allogeneic cells administered concurrently with immunosuppression or anesthesia were superior in aiding functional recovery, although autologous cells were found to offer greater benefit for structural repair. Interestingly, neither dosing nor timing of intervention was assessed as critical to functional improvement (9). These findings illustrate how addressing consistency and quality of pre-clinical trials is necessary for selection of appropriate study design, therapeutic approaches and treatments to carry forward for assessment in human populations.

## Conclusion

Investigators seeking to select a suitable animal model of stroke should consider their research hypothesis as different models are appropriate according to whether, e.g., ischemic insult or reperfusion injury is the focus. Rodent models are recommended by international authorities and combine similarity of cerebrovascular anatomy to humans with a practical handling size, but genetic manipulation studies are most suitably achieved in mice (51, 61, 152). Particularly due to ethical considerations, judicious use of larger animal models is indicated, and in this regard the recent development of a sheep model, which is rarely a companion species, may offer promise (65). The emerging imaging techniques facilitate combination of non-invasive, high-resolution anatomical analysis with detailed, multi-parametric physiological mapping. Perfusion MRI alone can provide detailed information about the evolution of ischemic lesions, confirming the validity of models and the effect of possible revascularization interventions. Perfusion and diffusion MRI taken together deliver a good approximation of the ischemic penumbra, and therefore enable experiments to directly assess recovery of at-risk tissue in sub-chronical time-windows. In parallel, fMRI enables assessment of how reparative interventions can modulate the physiological re-organization processes that take place in the peri-lesional cortex, and at system-level. The currently available models and MR imaging techniques thus provide a robust platform for investigating possible reparative interventions. Despite much attention in past decades on stem cell therapy, human application remains in its early stages and benefits, particularly with exogenous therapies, are likely due to a positive bystander effect rather than the generation of functionally relevant synapses (171). Endogenous therapy is therefore a key research goal, but is less advanced than exogenous strategies. Of these, while ASCs may be more readily extracted, a greater body of evidence exists for BM-derived stem cells (172). However, any approach should consider safety issues and the hypoxic and pro-inflammatory nature of the target tissue with MSC priming and concurrent neuroprotective therapy likely prerequisites of treatment (130, 133, 165, 172).

## Conflict of Interest Statement

The authors declare that the research was conducted in the absence of any commercial or financial relationships that could be construed as a potential conflict of interest.
